# Epidemiology, presentation and population genetics of patent ductus arteriosus (PDA) in the Dutch Stabyhoun dog

**DOI:** 10.1186/s12917-016-0720-x

**Published:** 2016-06-13

**Authors:** Marjolein L. den Toom, Agnes E. Meiling, Rachel E. Thomas, Peter A. J. Leegwater, Henri C. M. Heuven

**Affiliations:** Department of Clinical Sciences of Companion Animals, Faculty of Veterinary Medicine, Utrecht University, Yalelaan 108, 3508 TD Utrecht, The Netherlands; Department of Pathobiology, Faculty of Veterinary Medicine, Utrecht University, Yalelaan 1, 3485 CL Utrecht, The Netherlands; Animal Breeding and Genomics Centre, Wageningen University, P.O. box 338, 6700 AH Wageningen, The Netherlands

**Keywords:** Canine, Genetics, Heritability, Patent ductus arteriosus, PDA, Prevalence, Sex predisposition

## Abstract

**Background:**

Patent ductus arteriosus (PDA) is one of the most common congenital heart defects in dogs and is considered to be a complex, polygenic threshold trait for which a female sex predisposition has been described. Histological studies in dogs suggest that smooth muscle hypoplasia and asymmetry of the ductus tissue is the major cause of PDA. The Stabyhoun population is small and a predisposition for PDA has been suggested. The aims of this study were to describe the incidence, presentation from a clinical and histopathological perspective, and the population genetics of PDA in the Dutch Stabyhoun population.

**Results:**

Forty-six cases were identified between 2000 and 2013. Between 2009 and 2012 the birth incidence of PDA in the Stabyhoun breed was 1.05 %. We estimated this to be 7–13 times higher than expected in the general dog population. Twelve of the 46 cases were part of a litter in which more than one sibling was affected. There was no sex predilection in our case cohort. Dogs diagnosed in adulthood showed severe cardiomegaly. The mean inbreeding coefficient of the reference population of Stabyhoun dogs was 31.4 % and the actual and effective numbers of founders were 14 and 6.5, respectively. The heritability of PDA was 0.51 (±0.09) for the reference population and 0.41 (±0.10) for the phenotyped population. Histopathology of sections of the PDA from two dogs showed findings similar to those described in other breeds although the smooth muscle of the ductus adjacent to the pulmonary artery appeared more hypoplastic than that in the ductus adjacent to the aorta.

**Conclusions:**

The Stabyhoun breed shows a strong predisposition for PDA. Apart from the absence of a higher incidence in females, no other significant features distinguish PDA in Stabyhouns from the condition in other dog breeds. Heritability and the mean inbreeding coefficient are both very high making the Dutch Stabyhoun breed particularly suited to the study of inherited risk factors for PDA.

## Background

Patent ductus arteriosus (PDA) is a condition in which the ductus arteriosus (DA), also known as the *ductus Botalli*, fails to close after birth. During foetal growth the DA carries blood from the pulmonary artery to the aorta, to bypass the fluid-filled, not yet functioning lungs. In dogs, the DA closes within the first week of life [1] by contraction of the DA muscle, enabling normal blood flow through the lungs and aorta. Before birth prostaglandin E_2_ (PGE2) from the placenta maintains the patency of the DA by relaxing the DA muscle [2]. When the lungs open, oxygen tension rises in the blood, inducing closure of the DA through the endothelin signalling pathway [2]. In cases of PDA, however, the DA fails to close, causing a shunt through which blood flows in a pattern that deviates from the normal route of the circulatory system. The direction of the abnormal blood flow is determined by the left and right heart pressures (“left to right”, i.e. systemic to pulmonary or “right to left”, i.e. pulmonary to systemic). At birth, all shunts are “left to right”, due to the higher pressures in the left heart. However, over time a complication known as pulmonary hypertension can develop. If the pulmonary hypertension is severe enough, the direction of blood flow through the shunt can reverse into a “right to left” shunt. This “reversal” occurs in approximately 5 % of dogs with PDA [3]. Blood flowing from the aorta into the pulmonary artery (left to right shunt) typically causes a continuous heart murmur, increased pulmonary vascularity, left atrial and left ventricular enlargement and dilation of the aortic arch. If untreated, most dogs develop left heart failure within the first year of life [4, 5]. In dogs with reversed PDA, the murmur disappears and dogs will show cyanosis, secondary polycythemia and hyperviscosity. This can lead to exercise intolerance, intermittent hind limb weakness and seizures [6, 7]. Some breeds of dog appear to have a strong predisposition for this defect [8] and inheritance has been clearly demonstrated in the Miniature Poodle [9]. Different modes of inheritance for PDA have been proposed [8, 9], but it is currently accepted that hereditary PDA in the dog is not a simple Mendelian trait. A study demonstrated that the defect has a graded phenotypic expression and that more severely affected parents had more severely affected progeny [9]. A high proportion of the offspring of test matings had a fully patent ductus arteriosus, while a smaller proportion had a blind diverticulum of the ductus arteriosus which communicated with the aorta, but not with the pulmonary artery. This is considered to be a *forme fruste* of PDA, representing incomplete closure. Based on these findings, it was concluded that PDA resembles a quasi-continuous or threshold trait with a high degree of heritability and with the likelihood of defective closure of the ductus arteriosus being increased with the proportion of the genome received from dogs with PDA [9]. The population of Stabyhoun dogs, a Dutch hunting breed, is small (approximately 5500 animals worldwide) and seems to be predisposed to PDA. In a retrospective study, evaluating PDA in a referral population, the number of presented PDA positive Stabyhoun dogs was approximately 15 times higher than expected [10].

PDA is the second most common type of congenital heart disease in man with an estimated incidence of approximately two to eight cases per 10,000 births in full-term infants whilst it accounts for the majority of all cases of congenital heart disease in preterm infants [11, 12]. Two retrospective twin studies have suggested a familial component to PDA in preterm infants [13, 14]. Familial clustering of PDA has also been reported in full-term infants [15]. In human patients causative mutations have been found in a gene encoding smooth muscle myosin heavy chain *(MYH11)* [16] and a gene encoding transcription factor AP-2 beta *(TFAP2B)* [17, 18], found in neural crest derived cells. However, many human cases with a suspected genetic background remain unsolved [19]. In dogs, a relationship between prematurity and PDA has not been described. Histological studies of PDA's in dogs suggest that abnormal tissue architecture (mainly smooth muscle hypoplasia and asymmetry) is the major cause of PDA [20]. As PDA is one of the most common congenital heart defects in dogs and recent studies suggest a more important role for genetic factors in the occurrence of PDA in human infants, research into the etiology and inherited risk factors in dogs and humans has gained more interest. We propose that the Stabyhoun dog is a good model for further research. The aims of this study were to describe the incidence, presentation from a clinical and histopathological perspective, and the population genetics of PDA in the Dutch Stabyhoun dog population.

## Methods

### Animals and incidence

Records from the database of the the Dutch Kennel Club (Raad van Beheer) and the Utrecht University Companion Animal Clinic for the period 2000 to 2013 were evaluated. Forty-six cases of PDA were identified and included in this study and the recorded data were reviewed retrospectively. This included, when available, age at diagnosis, gender, number of affected siblings, direction of blood flow in the shunt, and presence of cardiomegaly.

The birth incidence for the three years from 2009 to 2012 was calculated from the number of known PDA positive cases and the total number of dogs born in this period.

To be able to include dogs in an “unaffected phenotyped” population, dogs were examined by a resident or board certified diplomate in veterinary cardiology on a yearly breeders day of one of the Dutch Stabyhoun breeding clubs (Nederlandse Vereniging voor Stabij-en Wetterhounen (N.V.S.W.). A dog was included in the unaffected phenotyped population if the dog was asymptomatic and did not show any abnormalities on cardiovascular examination.

### Histopathology

Histology of the PDA was performed in two puppies by one of the authors (RT) under supervision of a board certified pathologist. Results were compared to the described histological presentation in other breeds [20]. Samples were trimmed to obtain a transverse section that includes a transverse to diagonal section of the ductus with the adjacent aorta and pulmonary artery attached to the PDA. Tissues were fixed in 10 % neutral buffered formalin, embedded in paraffin according to standard procedures and cut into 4 μm tissue sections. Sections were stained with hematoxylin and eosin and picosirius red with alcian blue to visualize type I collagen and glycosaminoglycan (GAG)-rich intercellular matrix. Immunohistochemistry for smooth muscle actin (α-SMA) was performed on subsequent sections. Briefly, mouse anti-smooth muscle actin antibody^1^ as the primary antibody and dog anti-mouse/biotin^2^ as the secondary antibody were used according to manufacturers’ instructions to demonstrate the presence of smooth muscle.

### Population genetics

Pedigree data from the Dutch Kennel Club (Raad van Beheer) were retrieved for the period 1940 to 2013. For each dog the database records contained: registration number, registration number of both parents, date of birth, and sex. In the pedigree analysis, Stabyhoun dogs born from 2000 to 2013 were used as a reference population (in this study referred to as “REFPOP”). PDA positive dogs are referred to as “POSDOGS”. Pedigree files were analyzed with the Pedig package [21] and the following programs of this package were used: verification of the pedigree was successfully performed with *verif_ped*, checking for individuals present in their own pedigree; *Prob_orig* was used to determine both the actual number of founders and the effective number of founders by use of the classical approach [22]; *Meuw* was used to calculate the inbreeding coefficient for all individuals with the algorithm of Meuwissen and Luo [23]. To find common ancestors of all PDA positive dogs *anc_comm* was used. The percentage of contribution to each individual by the common ancestors was compared to the contribution of those individuals to the reference population. The total numbers of males and females were compared with the numbers of sires and dams used for breeding. Also, the maximum number of offspring per individual was assessed for both sires and dams.

### Statistical analysis

Heritability and variance components (σ^2^) were calculated using ASReml software [24]. Because of the proposed complex polygenic threshold trait [9], the following logistic model was applied to estimate the genetic and environmental variance, which were used to calculate heritability: y = μ + animal + e where μ is the overall mean. Animal is a random effect. Normal distributions are assumed for the random effects: animal ~ N (0, Aσ^2^_a_), where A contains the additive genetic relationship between animals. The residual variance (σ^2^_e_) was fixed at 3.289 in order to scale the animal effects. The heritability was calculated using the formula: h^2^ = σ^2^_a_/(σ^2^_a_ + σ^2^_e_) where σ^2^_a_ is the additive genetic variation and σ^2^_a_ + σ^2^_e_ is the phenotypic variation. Variance components (including relationship matrices) and heritability were estimated in two datasets. Once over the entire REFPOP, assuming all individuals that did not have a known PDA were healthy, and once over the phenotyped population: the population of Stabyhoun dogs that were confirmed to be either PDA positive (*n* = 46) or PDA negative, based on clinical examination by a resident or board certified diplomate in veterinary cardiology (*n* = 213). For significance testing the software PASW Statistics 18 was used.

## Results & discussion

### Incidence

Between 2000 and 2013, 46 cases of PDA were identified in this study population. Twenty-nine cases were born between 2009 and 2012 and in total over the same period, 2749 pups were born and registered. Based on this, the minimal birth incidence in the Stabyhoun breed was 1.05 % during this period. In a large retrospective study performed in a university clinic, the prevalence rate for all combined cardiovascular malformations in dogs, was 0.68 % [8]. In reality we expect this number to be lower, as animals referred to a university clinic are usually not representative of the general population. The percentage of PDAs amongst these congenital cardiac defects is estimated to be between 11 and 28 % [7, 8, 25, 26]. Therefore, we estimated the incidence of PDA to be lower than 0.08–0.14 % in the overall dog population and our results show the incidence in the Stabyhoun dog is approximately 7.5–13 times higher. Furthermore, the actual birth incidence may be even higher, as it is possible that not all cases are reported. For example, as PDA can cause death at a very young age [8] necropsy is often not performed in these dogs. In addition, when PDA is diagnosed, cases are not always reported to the breeders associations and/or Dutch Kennel Club.

### Clinical presentation

Of the 46 PDA positive dogs, 12 dogs were part of a litter in which more than one dog was known to be affected. We recorded three litters with two affected siblings and two litters with three affected siblings. Mean age at diagnosis was 316 days with a range of 39–2865 days. Most dogs were diagnosed as young pups, but four dogs were diagnosed as adults (14, 21, 52 and 96 months old). These four dogs were still asymptomatic but showed cardiomegaly at the time of diagnosis. Only one dog, aged approximately six months at presentation, was diagnosed with a “reversed” PDA. Twenty-three dogs were male and twenty-three dogs were female. Apart from the absence of a higher incidence in females, no other significant features distinguish PDA in Stabyhouns from the condition in other dog breeds. In both canine and human studies a clear predisposition for females has been reported [8, 10, 26, 27]. The reason for this female predisposition is unclear. Surprisingly, no sex predilection was seen in this case cohort. The reason for the absence of a female predisposition in the Stabyhoun breed is unclear as the number of animals should be sufficient to show a significant difference if present. This could be an important distinguishing feature of PDA in this breed and further studies are necessary to further investigate this phenomenon.

### Histopathology

**Fig. 1:**
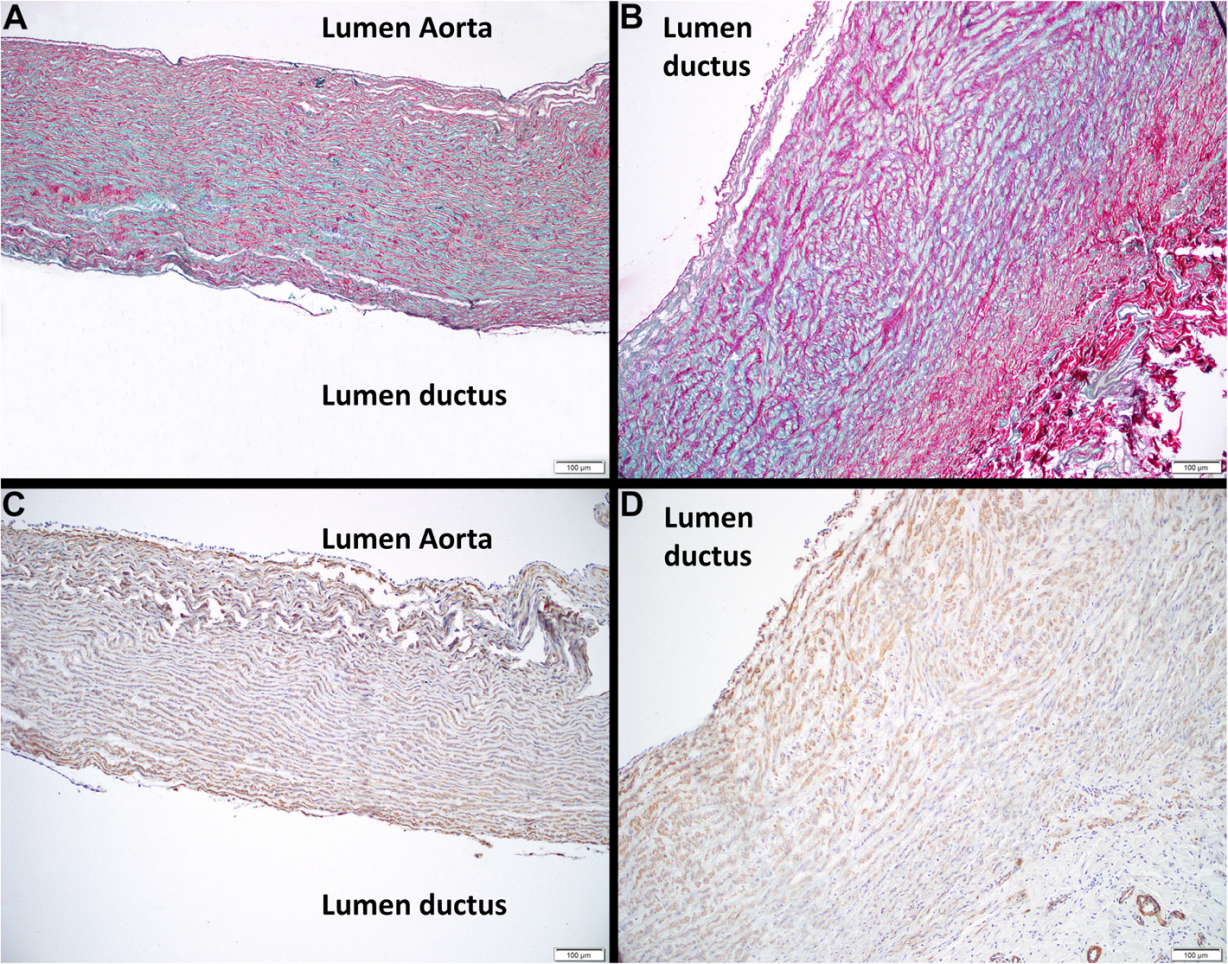
Histopathology of PDA in Stabyhoun dogs. **a** Picosirius red with Alcian blue: Wall of the persistent ductus arteriosus adjacent to the aorta showing approximately evenly distributed collagen and GAG-rich extracellular matrix. ×100. **b** Picosirius red and Alcian blue: Wall of the PDA adjacent to the pulmonary artery; the collagen is irregularly arranged with short crisscrossing bundles separated by irregular deposits of GAG-rich extracellular matrix. ×100. **c** Smooth muscle actin: Wall of the persistent ductus arteriosus adjacent to the aorta with immunohistochemical staining of smooth muscle actin highlighting the concentric and relatively even distribution of smooth muscle cells. ×100. **d** Smooth muscle actin: Wall of the persistent ductus arteriosus adjacent to the pulmonary artery; bundles of smooth muscle cells are short and irregularly distributed. ×100

The ductus arteriosus in both puppies was approximately 0.5 cm in length with a lumen of 0.5–1 cm. The tissues of both puppies have similar histological characteristics. The wall of the pulmonary artery is composed of little collagen and consists predominantly of smooth muscle and GAG-rich extracellular matrix. The type I collagen in the aortic wall is regularly, circumferentially distributed between smooth muscle cells. In general the type I collagen in the wall of the PDA appears to be less densely organized than that in the walls of the aorta and pulmonary artery. In the wall of the PDA adjacent to the aorta the collagen fibers are arranged concentrically and separated by evenly distributed GAG-rich extracellular matrix (Fig. 1a). In the wall of the PDA adjacent to the pulmonary artery the collagen is irregularly arranged with short crisscrossing bundles separated by irregular deposits of GAG-rich extracellular matrix (Fig. 1b). Similar to the collagen bundles, the smooth muscle cells are also more haphazardly organized and appear less dense in the side of the PDA adjacent to the pulmonary artery (Fig. 1c and d). The histological results from these dogs show some agreement with the results described by Buchanan et al. [20], however there are also some differences which may reflect the differences in the technique used for histological assessment: Whereas Buchanan made a quantitative analysis over the whole length of the PDA our images are from a small number of sections. In the cases presented here, the structure of the wall of the PDA in both puppies is asymmetric in terms of the organization of the distribution of collagen and smooth muscle fibers with regards to one another and the GAG-rich component of the extracellular matrix. However, as only a single site was analyzed, it is possible that these differences reflect differences in depth and angle of sectioning. Future studies could analyze the wall structure of non-persistent ductus arteriosus in age-matched Stabyhoun control dogs.

### Population genetics

#### Pedigree structure

The entire evaluated Stabyhoun pedigree consisted of 14,955 dogs, bred between 1940 and 2013. In the reference population (REFPOP) from 2000 to 2013, 7008 dogs were born. The number of males did not differ significantly from the number of females in both the entire (*p* = 0.139) and reference populations (*p* = 0.173). Additionally, equal numbers of males and females were affected with PDA (POSDOGS; 23 males and 23 females). The number of founders in the studbook for the entire population is 38. The actual number of founders for the reference population was 14. In other words, all individuals born between 2000 and 2013 descended from a total of 14 individuals. The effective number of founders was lower, only 6.5. This indicates that in an idealized population, were all matings are at random, only 6.5 founders would give the same level of inbreeding. The percentage of females that are mothers was 22.2 % in the REFPOP, whereas the percentage of males that are fathers was only 11.8 %. Over the entire population the percentage of females that are mothers was 15.1 %, which is lower than 22.2 %, indicating that more females were used for breeding in the current population compared to before 2000. The same is true for males, where only 6.8 % fathered at least one litter, over the entire population, compared to 11.8 % in the REFPOP. The maximum number of offspring per sire was 392 for the entire population and 127 for the REFPOP. Per dam the maximum number of offspring was 78 for the entire population and 52 for the REFPOP.

#### Inbreeding coefficients

There was no significant difference in inbreeding coefficient (F) between the REFPOP and the POSDOGS. The mean inbreeding coefficient of the REFPOP was 31.4 % and the mean inbreeding coefficient of the POSDOGS was 31.5 %. Results of the pedigree structure analysis and inbreeding coefficients are summarized in Table 1. Both inbreeding coefficients are higher than the inbreeding coefficient of a brother-sister or parent-offspring mating, which give the highest level of inbreeding in a single generation (25 %). This is notably higher when compared to other dog breeds [28–30]. However, for most of the breeds in these studies not all known ancestry data is taken into account. In this study we analysed approximately 12 generations of Stabyhoun dogs, since the recognition of the breed in 1942. In other studies, records were mainly reviewed from the 1970 – 1990's and they generally assumed that all individuals present at that time were nonrelated. However, in three scent-hound breeds from the alpine region, pedigrees since founding in the 1930s were investigated. In these breeds, after correcting for pedigree completeness, inbreeding coefficients of 6.4, 9.1 and 13.6 % for Bavarian mountain hound (BMH), Hanoverian hound (HH) and Tyrolean hound (TH), respectively, were demonstrated [31]. The BMH has a similar population size to the Stabyhoun, whereas HH and TH have significantly smaller populations. As population size is a strong predictor for inbreeding coefficients, it could be expected that the Stabyhoun and BMH would have similar inbreeding coefficients, but the Stabyhoun population shows a higher level of inbreeding. This can be explained by the low number of founders for the Stabyhoun population. Although the Stabyhoun studbook contains 38 potential founders, only 14 of these founders are actual ancestors of the current day population (the REFPOP) and the effective number of founders is 6.5. The scent-hound breeds had actual numbers of founders of 353 (BMH), 226 (HH) and 98 (TH) and effective numbers of founders of 43.7 (BMH), 41.6 (HH) and 18.9 (TH) [31]. This is considerably higher and facilitates the avoidance of inbreeding. A French study examining all members of nine different dog breeds that were registered between 1975 and 2001 reported founder numbers of 13 (Barbet) to 1158 (Epagneul Breton) and corresponding effective founder numbers of 6.9–70.7 [28]. The effective number of founders of the Stabyhoun is below this range, whereas the actual number of founders is within the range. Breeding practices that cause uneven contribution of founders to the REFPOP, such as the use of popular sires, may underlie this discrepancy. In the Stabyhoun breed there are indications of the use of popular sires, as only 11.8 % of males father at least one litter. In addition, within the entire population one particular sire fathered 392 offspring. For the current day population the maximum number of offspring for one sire is 127, approximately corresponding to 18 litters for a single male. The maximum number of offspring per sire and dam has decreased over time.

**Table 1:** Genealogical parameters for PDA positive cases, reference population and total of the Stabyhoun population

	POSDOGS	REFPOP	Total
N	46	7008	14,955
F mean	0.315	0.314	0.268
F max	0.360	0.389	0.466
Proportion *F* > 0.1	1.0	1.0	0.951
Founders	–	14	38
Effective # founders	–	6.5	–
# males	23	3424	7387
# sires	–	403	503
% of males that sire	–	11.8	6.8
Max. offspring per sire		127	392
# females	23	3541	7568
# dams	–	787	1139
% of females that sire	–	22.2	15.1
Max offspring per dam		52	78

*POSDOGS:* PDA positive cases, registered at databases of the Dutch Kennel Club and/or the Utrecht University Companion Animal Clinic between 2000 and 2013. *REFPOP*: the total reference population of Stabyhoun dogs born from 2000 to 2013 and registered in database of the Dutch Kennel Club. *N: * number of individuals, *F mean:* mean inbreeding coefficient, *F max:* maximal inbreeding coefficient, proportion *F* > 0.1: the proportion of dogs in the population with an inbreeding coefficient higher than 0.1

#### Heritability

The heritability of PDA was calculated over the entire reference population, assuming all individuals that did not have a known PDA status were healthy, and over the phenotyped population. Using an animal model the variance components for animal effects were 3.40 for REFPOP and 2.24 for the phenotyped population. The heritability of PDA was 0.51 (±0.09) for the reference population and 0.41 (±0.10) for the phenotype population. Results of the heritability analysis are summarized in Table 2. In both cases the heritability differed significantly from zero, showing a high hereditary component to PDA in the Stabyhoun dog.

**Table 2:** Variance components and heritability of PDA in Stabyhoun dogs

	σ^2^_a_	σ^2^_e_	σ^2^_total_	h^2^	SE (h^2^)
REFPOP	3.40	3.29	6.69	0.51	0.09
Phenotyped	2.24	3.29	5.53	0.41	0.10

*PDA:* Patent ductus arteriosus, *REFPOP:* the total reference population of Stabyhoun dogs born between 2000 to 2013 and registered in database of the Dutch Kennel Club (*n* = 7008), *Phenotyped*: the population of Stabyhoun dogs that were confirmed to be either PDA positive (*n* = 46) or PDA negative, based on clinical examination by a resident or board certified diplomate in veterinary cardiology (*n* = 213), *σ* ^*2*^_*a*_: the additive genetic variance, *σ* ^*2*^_*e*_: the residual variance, *σ* ^*2*^_*total*_ (σ^2^_a_ + σ^2^_e_): the phenotypic variance, *h* ^*2*^: narrow sense heritability, *SE (h* ^*2*^ *): *standard error of narrow sense heritability

#### Common ancestors

Only one dog showed a significantly higher percentage of genetic contribution to the PDA cases compared to the reference population. However, this individual, coded as individual 1212 in our analyses, also contributed to all individuals in the reference population (REFPOP) to some degree. The genetic contributions of the seven most influential dogs are illustrated in Table 3. One of the limitations of this study is the fact that dogs were categorized either as positive (confirmed PDA) or negative (absence of continuous murmur and/or clinical signs). However, as described earlier, some dogs might have a blind diverticulum instead of a full PDA. This does not cause a murmur or clinical signs and, therefore, is likely to go unnoticed as it can only be diagnosed by post-mortem examination or aortic angiography. As aortic angiography was not performed in the phenotyped population the presence of blind diverticulums in some dogs considered as negative in our analysis cannot be excluded. Our pedigree analysis confirms that the inheritance of PDA is not Mendelian, with low numbers of affected sibling pairs and lack of parent-offspring transmission.

**Table 3:** The genetic contribution of most influential ancestors to positive cases and the reference population

Identification number	POSDOGS	REFPOP	Difference
1212	5.0	4.3	0.7*
411	5.0	4.8	0.2
261	28.5	28.4	0.1
72	41.5	41.5	0
196	28.9	28.9	0
71	24.5	24.6	−0.1
25	19.5	19.6	−0.1

The percentage of genetic contribution of the seven most influential ancestors is demonstrated for the PDA positive cases (POSDOGS) and the reference population (REFPOP). *significant difference (*p* = 0.000). REFPOP: Stabyhoun dogs born from 2000 to 2013 and registered in database of Dutch Kennel Club (*n* = 7008). POSDOGS: PDA positive cases, registered at databases of the Dutch Kennel Club and/or the Utrecht University Companion Animal Clinic between 2000 and 2013 (*n* = 46). Note that there is only one common ancestor to the PDA positive cases that significantly contributes more to the positive individuals than to the reference population

## Conclusions

In conclusion, the results of the present study suggest that the Stabyhoun dog is a breed with a strong predisposition for PDA. Apart from the absence of a higher incidence in females, no other significant features distinguish PDA in Stabyhouns from the condition in other dog breeds. Heritability and the mean inbreeding coefficient are both very high and we, therefore, propose that the Dutch Stabyhoun breed is particularly suited to the study of inherited risk factors for PDA.

## Abbreviations

BMH, Bavarian mountain hound; HH, Hanoverian hound; PDA, patent ductus arteriosus; *PHENOTYPED,* the population of Stabyhoun dogs that were confirmed to be either PDA positive (*n* = 46) or PDA negative (*n* = 213), based on clinical examination by a resident or a board certified diplomate in veterinary cardiology; *POSDOGS,* PDA positive cases, registered at databases of the Dutch Kennel Club and/or the Utrecht University Companion Animal Clinic between 2000 and 2013 (*n* = 46); *REFPOP*, the total reference population of Stabyhoun dogs born between 2000 to 2013 and registered in database of the Dutch Kennel Club (*n* = 7008); TH, Tyrolean hound.

## Footnotes

1 Biogenex, Fremont, California, United States of America.

2 Vector Laboratories, Peterborough, United Kingdom.
